# The draft genome dataset of the American cockroach, *Periplaneta americana* (Linnaeus, 1758) (Blattidae: Blattinae)

**DOI:** 10.1016/j.dib.2023.109301

**Published:** 2023-06-07

**Authors:** Marcellinus Isaac Stia Dominic, Abdul Hafiz Ab Majid

**Affiliations:** a Household & Structural Urban Entomology Laboratory, School of Biological Sciences, Universiti Sains Malaysia, Penang 11800, Malaysia; b Centre for Insect Systematics (CIS), Faculty of Science and Technology, Universiti Kebangsaan Malaysia (UKM), Bangi 43600, Malaysia

**Keywords:** *Periplaneta americana*, American cockroach, Genomic DNA, Whole-genome sequencing

## Abstract

*Periplaneta americana* is a cosmopolitan pest cockroach endemic to tropical and subtropical climates. It occurs frequently in urban sewer and wastewater system and transit in human proximities, spreading pathogens that causes serious public health concerns such as asthma, allergies, and others. By using the Next-generation Sequencing (NGS) known as Illumina NovaSeq 6000, this article documents for the draft genome data set of *P. americana* collected in Penang Island, Malaysia. This article displays the pair-end 150 bp genome dataset and results on the sequence quality. This genome dataset presents the information for further understanding of *P. americana* populations at molecular level and the opportunity to develop effective control and management strategies for the species. This dataset is available under Sequence Read Archive (SRA) databases with the SRR23867103.

**Specifications Table**

**Table:** 

Subject	Biological Sciences
Specific subject area	Entomology and insect science, Genomics: Next generation sequencing (NGS) reads of genome
Type of data	Figures and raw reads of sequenced genome
How the data were acquired	Paired end reads of *P. americana* genome were sequenced using Illumina NovaSeq 6000
Data format	Raw sequencing data: FASTQ file.
Description of data collection	*Periplaneta americana* was baited using a baited glass jar with beer-soaked bread. DNA was extracted using HiYield Plus^TM^ Genomic DNA Mini Kit (Blood/tissue/cultured cells) (Real Biotech Corp, Taipei, Taiwan).
Data source location	· Institution: Universiti Sains Malaysia · City/Town/Region: Penang · Country: Malaysia · GPS coordinate for the collected samples: N 5° 21′ 24.67660′′, E 100° 17′ 58.62320′′
Data accessibility	BioProject: PRJNA944812 BioSample: SAMN33762737 Repository name: NCBI SRA (SRR23867103) Annotation Data: https://figshare.com/articles/dataset/Functional_Annotations/23211494/2 Direct URL to data: https://www.ncbi.nlm.nih.gov/bioproject/PRJNA944812

## Value of the Data

• *Periplaneta americana* represents a pest species endemic to tropical and subtropical climates and can be found inhabiting in urban areas, especially in sewers.
• Results obtained are particularly useful for urban and medical entomologist in Malaysia.
• The *P. americana* genome data could be used for the development of species-specific microsatellite marker.
• Further study could potentially contribute to pest control and management approach according to genetic diversity of *P. americana*.

## 1 Objective

The American cockroach, or also known as *Periplaneta americana* (*P. americana*), is predominantly found indoors in warm, humid regions. In addition, unlike German cockroaches which in general do not reproduce outside human structures, the American cockroaches has the capabilities to maintain their large populations in outdoor niches [1]. Particularly by which, the *P. americana* can reproduce in locations where there are accumulations of waters and of these, several studies implicated sewer systems as the main source of urban American cockroaches [2], [3]. This abundance in close proximity to urban areas has shown as a substantial threat to home allergens and human public health [4] similar to mosquito [5]. Despite this, however, the knowledge of the species is still limited due to the lack of genome-related data. To develop effective management strategies for sewer systems and control strategies for the species, it is crucial to study their urban populations at molecular level. Hence, this study aimed to draft the whole genome sequences of *P. americana* using Next-generation Sequencing (NGS) via Illumina NovaSeq 6000.

## 2 Data Description

This article described a dataset of whole-genome pair-end sequencing result of BioSample *SAMN33762737* which is under the BioProject *PRJNA944812*. This data is registered under the Sequence Read Archive (SRA) databases with the accession number *SRR23867103*. The data set comprised of two high throughput sequencing fastq files:

• PA1_DKDN220024683-1A_HNVCCDSX5_L4_1.fq
• PA1_DKDN220024683-1A_HNVCCDSX5_L4_2.fq

PA1_DKDN220024683-1A_HNVCCDSX5_L4_1.fq and PA1_DKDN220024683-1A_HNVCCDSX5_L4_2.fq contained half of the full sequence in a total of 36,359,668 raw reads with 150bp each. PA1_DKDN220024683-1A_HNVCCDSX5_L4_1.fq composed of the 1^st^-150^th^ base position meanwhile the 151^th^-300^th^ base position composed of from PA1_DKDN220024683-1A_HNVCCDSX5_L4_2.fq of each sequence.

**Fig. 1:**
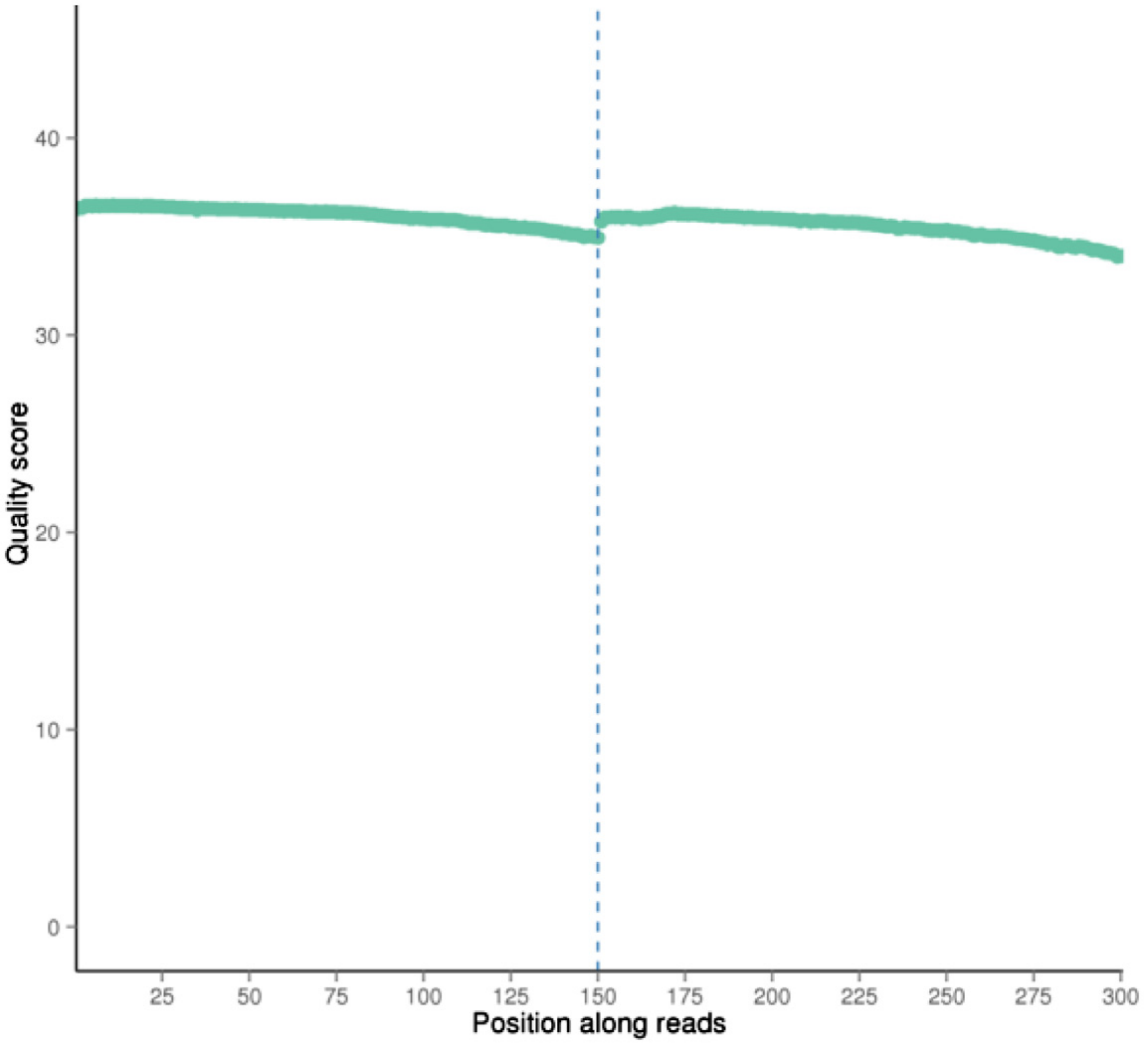
Quality score distribution along reads position.

**Fig. 2:**
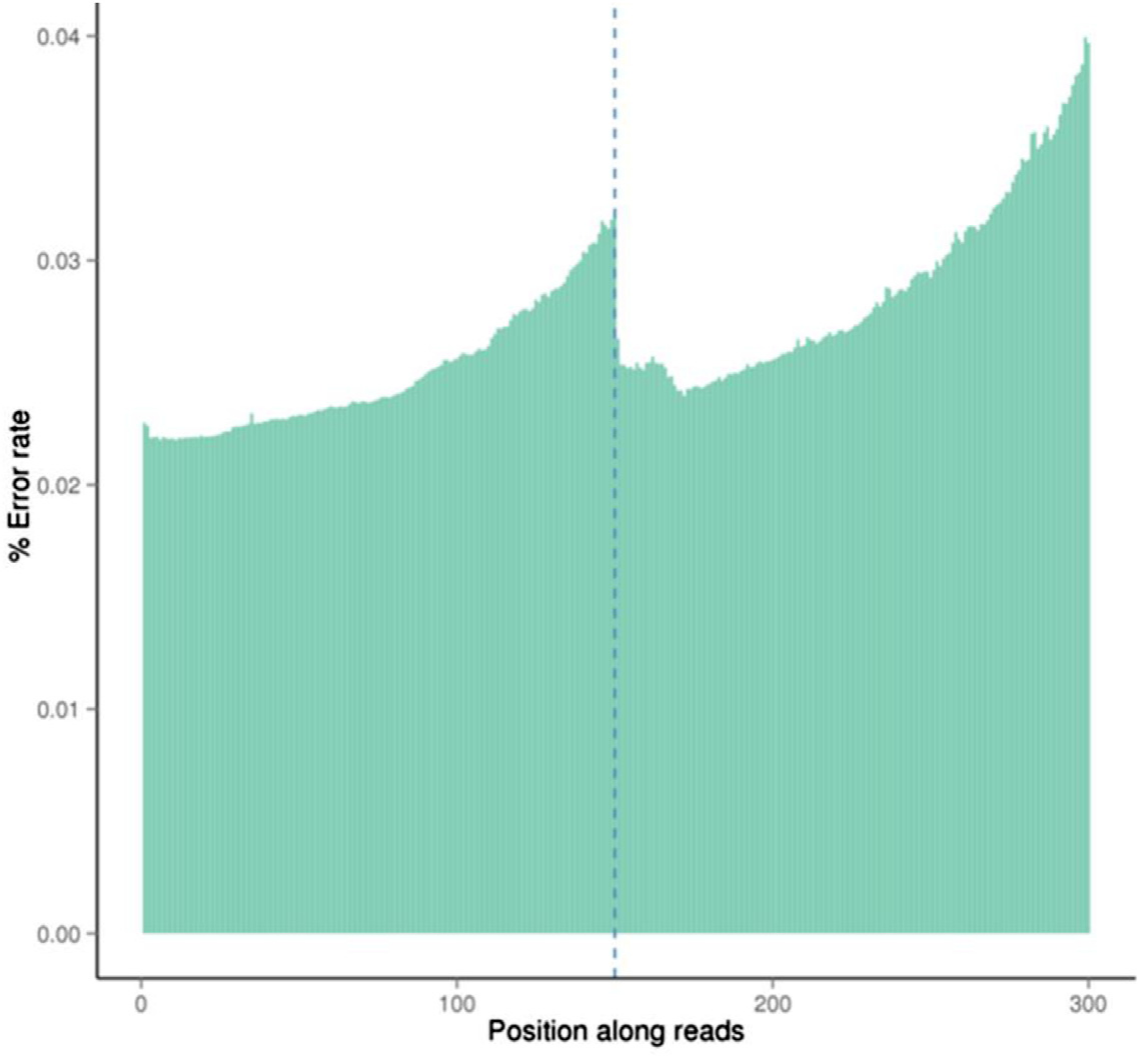
Error rate distribution along reads position.

**Fig. 3:**
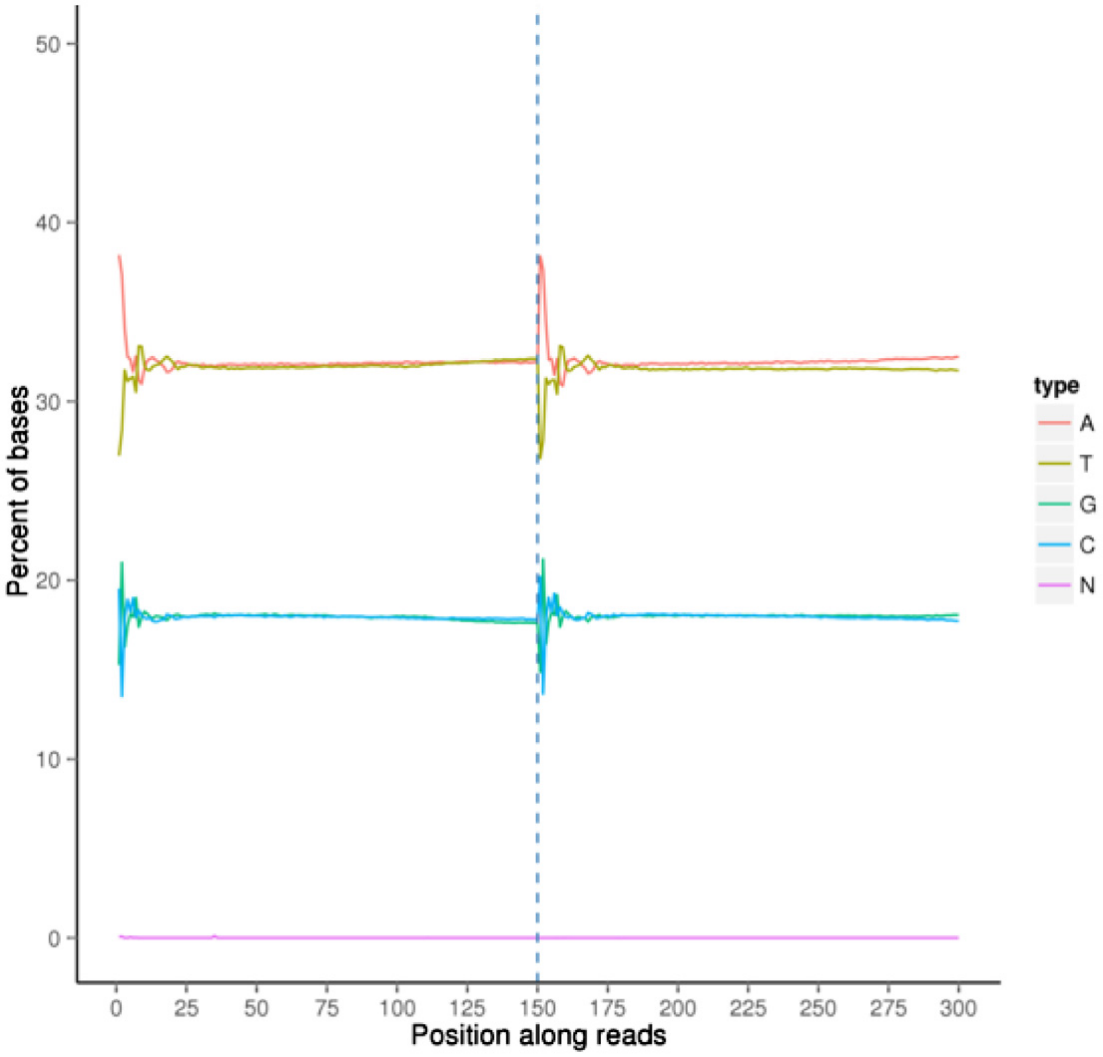
Bases content along reads position.

**Fig. 4:**
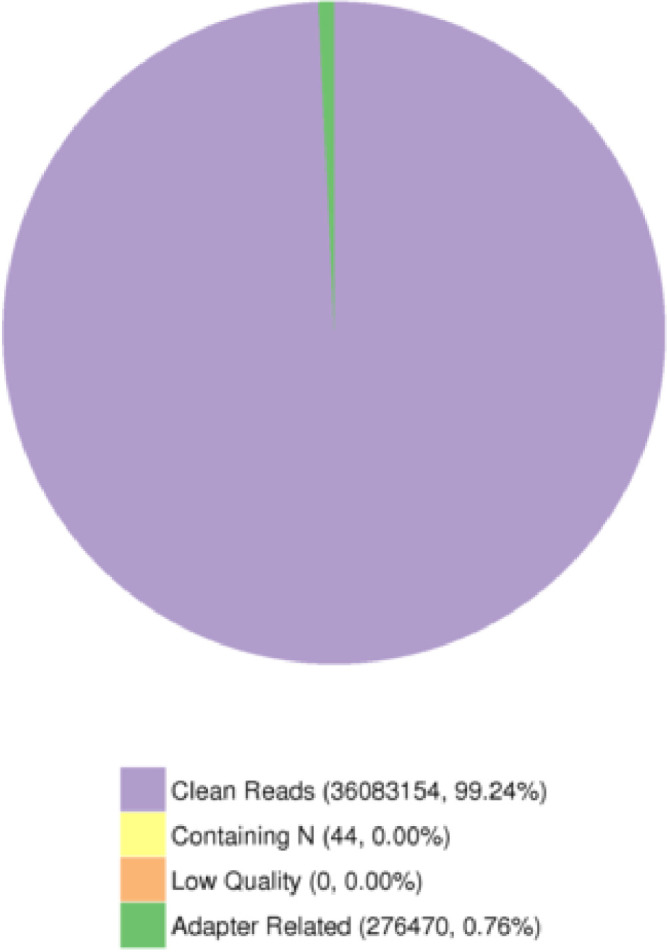
Classification of raw reads.

In this manuscript, characteristics and quality of dataset is presented. Firstly, the dataset's quality score scored between Q30 to Q40, whereby Q30 showed 99.9% of the correct base meanwhile Q40 showed 99.99% of correct base (Fig. 1). Secondly, the rate of the single base error rate distribution along reads is under 0.04% (Fig. 2). The total GC content represented at 36.69% (Fig. 3). Next, the sequencing data filtration can be seen that out of the 36,359,668 raw reads, in percentage, 99.24% are clean reads and 0.76% reads related to the adapter sequence (Fig. 4). Lastly, the forward adapter sequence is 5′-AGATCGGAAGAGCGTCGTGTAGGGAAAGAGTGTAGATCTCGGTGGTCGCCGTATCATT-3’, and the reverse adapter sequence is 5′-GATCGGAAGAGCACACGTCTGAACTCCAGTCACGGATGACTATCTCGTATGCCGTCTTCTGCTTG-3′.

## 3 Experimental Design, Materials and Methods

### 3.1 Sampling and DNA extraction

The American cockroach, *Periplaneta americana*, was collected using a baited glass jar with beer-soaked bread and was left in a sewer manhole shaft overnight from late evening till the next morning [4]. After collection, the *P. americana* was then freeze killed and stored in 95% ethanol under –20°C. An individual of *P. americana* was used for the genomic DNA (gDNA) extraction. The gDNA extraction was extracted solely from the leg tissue in order to reduce the possibility of DNA extraction contamination from endosymbionts [6]. The gDNA extraction was conducted using HiYield Plus^TM^ Genomic DNA Mini Kit (Blood/Tissue/Cultured Cells) (Real Biotech Corp., Taipei, Taiwan) according to the manufacturer's instruction. The leg tissues were vortexed by using MX-S Dragon Lab Single-head Vortex Mixer in lysis buffer with Proteinase K and incubated for 1 hour at 60°C by using MINIC-100 Mini Dry Bath. Elution was then be carried out twice using 50 µL elution buffers after the DNA binds to the filter column through an ethanol wash to get a total of 100 µL gDNA solution [7]. The extracted gDNA was then quantified by using OPTIZEN^TM^ NanoQ Lite Microvolume Spectrophotometer.

### 3.2 Library preparation and sequencing

**Fig. 5:**
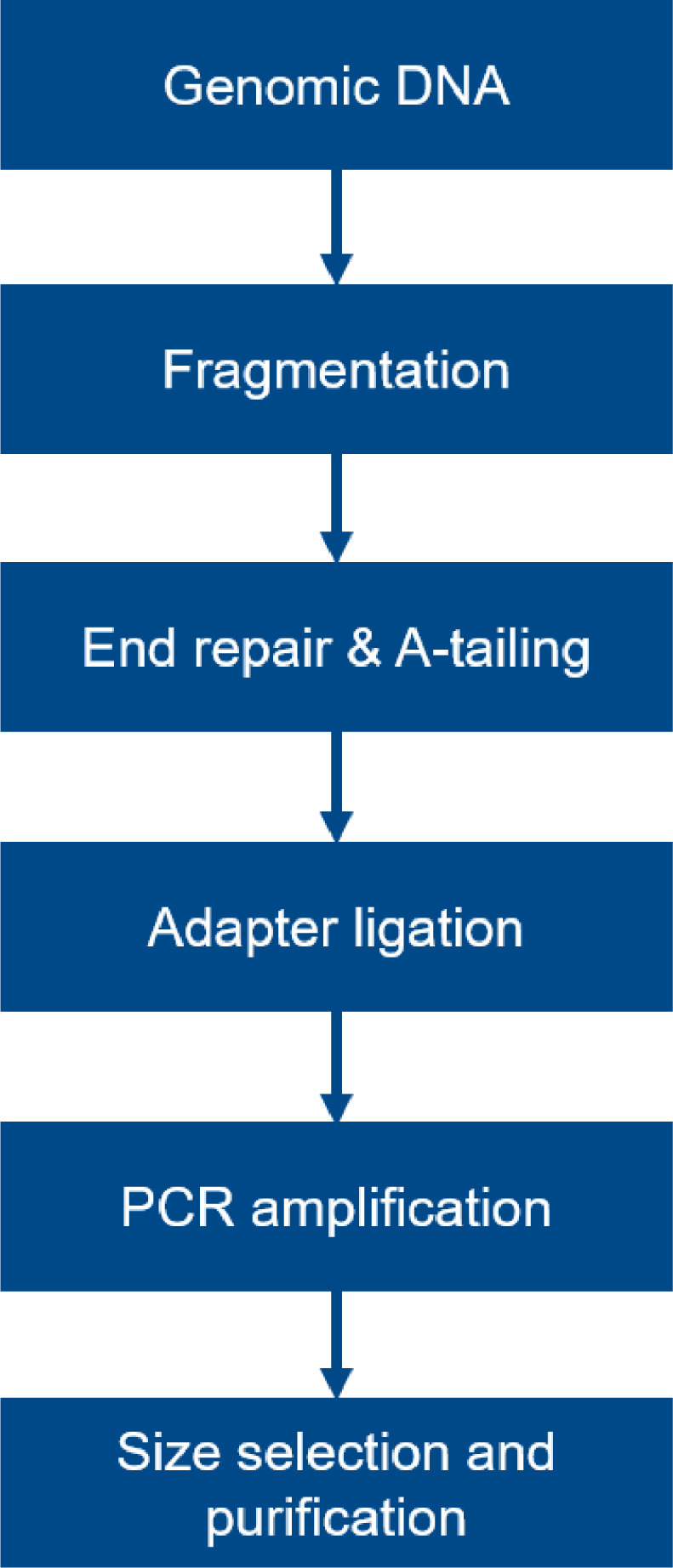
Workflow of library construction.

The general workflow of library construction is as shown in Fig. 5. The genomic DNA was randomly sheared into short fragments and the fragments obtained were then end repaired, A-tailed, and further ligated with Illumina adapters for sequencing. Next, the fragments with adapters were amplified, size selected, and purified.

Subsequently after that, the library was checked with Qubit and real-time PCR for quantification and bioanalyzer for size distribution detection. The quantified libraries will be pooled and sequenced on Illumina platforms, according to effective library concentration and data amount required [7].

### 3.3 Genome assembly

The Illumina sequencing data of *Periplaneta americana* were assembled, and an assembly with a total length of 15,064,661 bp and a contig N50 of 2089 bp was yielded (Table 1).

**Table 1:** Statistics of the *P. americana* genome assembly.

Assembly Statistics	SPAdes
No. contigs	48972
No. contigs (≥ 1000 bp)	2691
No. contigs (≥ 5000 bp)	148
No. contigs (≥ 10000 bp)	11
Total length (bp)	15,064,661
N50	2089
N90	743
L50	1049
L90	3566
GC (%)	36.69

### 3.4 Functional annotation of protein coding genes

Predicted protein sequences from this data were functionally annotated to EggNog mapper (Evolutionary Genealogy of Genes: Non-supervised Orthologous Groups) with a minimum E-value of 0.001 whereby 423 queries were scanned [8]. The functional annotation of genes was by performing mapping against public database, COG, the Clusters of Orthologous Groups (Table 2).

**Table 2:** Classification of the functional annotation of genes in the *P. americana* genome assembly based on the COG Database.

Category	Functions	Percentage (%)
BD	Jerky protein homolog-like	0.47
BQ	Semaphorin-plexin signaling pathway	0.24
D	Cell cycle control, cell division, chromosome partitioning	0.24
DT	Serine threonine-protein phosphatase	0.24
E	Amino acid transport and metabolism	28.13
G	Carbohydrate transport and metabolism	1.65
H	Coenzyme transport and metabolism	0.24
I	Lipid transport and metabolism	0.24
J	Translation, ribosomal structure and biogenesis	0.47
K	Transcription	0.95
L	Replication, recombination and repair	4.26
O	Posttranslational modification, protein turnover, chaperones	1.42
OU	K02A2.6-like	0.95
P	Inorganic ion transport and metabolism	0.95
Q	Secondary metabolites biosynthesis, transport and catabolism	0.24
S	No functional prediction	49.41
T	Signal transduction mechanisms	0.71
U	Intracellular trafficking, secretion, and vesicular transport	0.24
-	-	8.98

## Ethics Statements

Our work does not involve studies with humans. Our work involves studies with animals, specifically the American Cockroaches, *Periplaneta americana*.

## CRediT authorship contribution statement

**Marcellinus Isaac Stia Dominic:** Methodology, Investigation, Data curation, Formal analysis, Writing – original draft, Writing – review & editing. **Abdul Hafiz Ab Majid:** Conceptualization, Methodology, Supervision, Project administration, Resources, Funding acquisition, Writing – review & editing.

## Declaration of Competing Interest

The authors declare that they have no known competing financial interests or personal relationships that could have appeared to influence the work reported in this paper.

## Data Availability

Raw genome sequence of Periplaneta americana (Original data) (NCBI-GeneBank). Functional Annotations of American Cockroach, Periplaneta americana (Original data) (figshare.com).
